# Macroevolutionary patterning of woodpecker drums reveals how sexual selection elaborates signals under constraint

**DOI:** 10.1098/rspb.2017.2628

**Published:** 2018-02-21

**Authors:** Meredith C. Miles, Eric R. Schuppe, R. Miller Ligon, Matthew J. Fuxjager

**Affiliations:** Department of Biology, Wake Forest University, Winston-Salem, NC 27101, USA

**Keywords:** sexual selection, animal behaviour, signal design, constraint

## Abstract

Sexual selection drives elaboration in animal displays used for competition and courtship, but this process is opposed by morphological constraints on signal design. How do interactions between selection and constraint shape display evolution? One possibility is that sexual selection continues exaggeration under constraint by operating differentially on each signal component in complex, modular displays. This is seldom studied on a phylogenetic scale, but we address the issue herein by studying macroevolutionary patterning of woodpecker drum displays. These territorial displays are produced when an individual rapidly hits its bill on a hard surface, and drums vary across species in the number of beats included (length) and the rate of drumbeat production (speed). We report that species body size limits drum speed, but not drum length. As a result of this biomechanical constraint, there is less standing variation in speed than length. We also uncover a positive relationship between sexual size dimorphism and the unconstrained trait (length), but with no effect on speed. This suggests that when morphology limits the exaggeration of one component, sexual selection instead exaggerates the unconstrained trait. Modular displays therefore provide the basis for selection to find novel routes to phenotypic elaboration after previous ones are closed.

## 1. Introduction

Complex animal displays diversify in response to a tug-of-war between multiple evolutionary pressures. Most prominent is the conflict between constraints and selection. Here, constraints define boundaries that limit phenotypic exaggeration and standing variation [1], while selection elaborates displays by exaggerating existing components or by favouring the emergence of new display traits altogether [2,3]. Work on a proximate scale shows that sexual selection operates differentially based on the diverse constraints that influence signals [4–6], and thus it is an ongoing challenge to understand how this *micro*evolutionary process informs phenotypic patterning at the *macro*evolutionary scale. Although studies through the latter lens reveal that both constraint and selection profoundly influence display elaboration [7–9], the inherently complex nature of signal design suggests that these fundamental processes may work in unexpected ways [1,10].

In the current study, we explore this issue by testing how morphological constraints on a signal's production influence sexual selection for its design. Prior studies suggest that the solution may lie in the evolution of complex displays, which are constructed from more than one component signal or element [10–12]. Accordingly, complexity provides the phenotypic foundation for sexual selection to continue past the effects of constraint by allowing multiple signals to independently undergo modification [10]. This means that complex displays are modular: different signal components, or ‘modules’, each serve different functions and undergo their own evolutionary trajectory [10]. However, despite phenotypic and functional differences in each module, the full display still relies on the totality of all components rather than any one in isolation [11,13]. The interactions between multiple evolutionary drivers should therefore affect the macroevolutionary patterning of complex displays, but this topic is rarely addressed [11].

One of the main constraints on signal design is morphology [8,9]. Indeed, individual size differences limit the exaggeration of displays as diverse as crab claw-waves and spider seismic signals [5,14]. In these examples, small crabs cannot hold up a large claw to wave, and smaller spiders produce less intense vibrations on their web; thus, signal exaggeration (claw size or vibration amplitude) is constrained by body size. These effects are also apparent on a macroevolutionary basis, where morphological constraints restrict the potential for display divergence [8,9]. In other words, the primary consequence of constraint is a limit to the range of viable signal phenotypes. This may also influence other aspects of signal design, such as standing variation (the degree to which phenotypes vary within a species) [1,15]. This hypothesis remains untested in the macroevolutionary literature, but we predict that size-constrained signals should exhibit both (i) limited phenotypic space for exaggeration and (ii) reduced standing variation.

When a display consists of two elements—one that is not constrained by body size, and another that is—then which will sexual selection exaggerate? If constraints do indeed dictate standing variation, then sexual selection may favour the flexible signal, the constrained signal or both. For animal displays used in courtship and competition, each of these non-mutually exclusive trajectories is plausible. For instance, sexual selection may preferentially exaggerate unconstrained signals to preserve standing variation. This is possible because many animals rely on the ability to modulate their display performance according to social context (e.g. increasing display performance when confronting a threatening rival), which requires within-individual phenotypic variation [16–19]. If this is the case, constrained signals should be unrelated to indices of sexual selection, which will instead be positively correlated with more labile traits. The reverse could also be true, wherein constrained components are favoured instead. Considerable work focuses on this alternative, suggesting that sexual selection should operate on signals with restricted variation to make signals effective at conveying species or individual identity [20], or to honestly encode information about quality or condition [21]. Of course, these two directions for signal elaboration are non-mutually exclusive, because complex displays can consist of numerous components, each subject to a different combination of evolutionary pressures.

**Figure 1.**
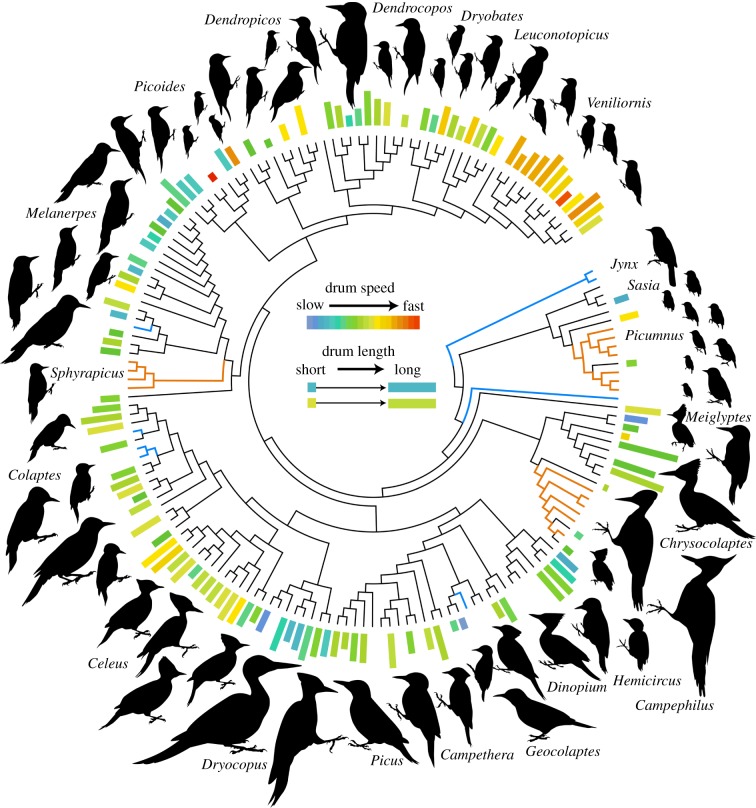
Cladogram of the woodpeckers (family: Picidae) from Dufort *et al*. [22]. At the tips, longer bars indicate species with longer drums. Bars are coloured by drum speed (beats s^–1^), with warm colours (green to red) indicating progressively faster drums than cool colours (blue-green to blue). Edge colors indicate the evolution of drumming determined from ER Mk1 ancestral state reconstruction (black, drum; orange, drum-like signal; blue, no drum or drum-like signal). Silhouettes are aligned near their corresponding tips, and drawn to-scale based on phylogenetic PCA of body size.

Here, we examine how constraint and sexual selection interactively shape animal displays by studying the macroevolutionary patterning of drumming behaviour in woodpeckers (Aves: Picidae), a widespread family of approximately 230 species [22]. Woodpeckers exhibit a wide range of body sizes, encompassing a 100-fold increase in size from the smallest to largest species (figure 1). Although these birds are well known for their innovative nesting and foraging strategies, a lesser-known woodpecker trait is their highly physical drum display, which serves as the main social signal during the breeding season [18,19]. To produce a drum, individuals rapidly and repeatedly hammer their bill against a hard substrate (typically a dead tree) in their environment, generating a loud sonation that is easily heard from afar. Woodpecker drums are the ideal display for this study for two main reasons. First, because drums are produced by striking one hard object against another, their acoustic frequency is characteristically broadband rather than tonal—sounding as distinct as a hand clap is from a whistle—which reduces the need to compute numerous frequency measures in order to achieve a biologically relevant measurement of display characteristics [23]. Instead, measurable variation in the drum display occurs in two ways: (i) cadence, or the patterning of beats over time and (ii) length, the number of beats in a drum [19]. To this end, there is considerable variation in both of these components of the drum, which provides ample grounds for evolutionary hypothesis testing (figure 1).

Second, previous studies suggest that drumming may be a modular signal influenced by both size constraints and sexual selection. Biomechanical models of drumming woodpeckers show that this gestural display can be extrapolated as a rod structure repeatedly traversing an angular distance to strike a stationary surface [24,25]. These models indicate that drum speed may be constrained by body size, as larger species must travel father to produce each drumbeat (electronic supplementary material, figure S1). However, there is no evidence that size would constrain drum length, which would make this display modular with regards to constraint. Drums also appear to be sexually selected for their primary use in territorial competition, as they are broadcasted actively during the breeding season deployed to drive off both conspecific and interspecific intruders [18,19]. Individuals are also able to distinguish between the drums of other species, which suggests that the signal facilitates conspecific mate choice by encoding species identity [19,26]. As such, female mate choice may influence drum elaboration, either via ‘adaptive’ mate choice for honest signals or ‘arbitrary’ preferences for signal aesthetics that convey no adaptive advantage [27,28]. Regardless of the mechanism, there are myriad opportunities for both constraint and sexual selection to influence drum design.

In this study, we use woodpecker drums to test, on a phylogenetic scale, how constraint and selection interact to shape the two primary components of a drum. We first investigate whether morphology constrains drum speed and length, while also testing for differences in standing variation between these two signal components. Then, we assess how sexual selection operates on these two signalling components by testing how sexual size dimorphism (SSD) predicts species differences in speed and length. SSD is a common index of sexual selection by male–male competition in many animals, and in birds it reflects the degree to which males compete for mates both directly and indirectly [29,30]. By examining the connection between SSD and signal design in constrained and unconstrained traits, we aim to uncover how sexual selection shapes complex displays in a modular fashion.

## 2. Material and methods

### (a) Acoustic data collection

We assessed the drum characteristics of 164 woodpecker species, spanning across all identified genera within Picidae. Woodpeckers use a drum as their primary territorial signal, which we operationally define as a mechanical sonation that consists of (i) greater than three beats and (ii) beats patterned over time at either a constant speed or constant acceleration of speed (see the electronic supplementary material, figure S2 for further explanation). This twofold definition is necessary because some woodpecker species produce drum-like signals that are not adequately described by a single average speed. As a result, we dropped 12 species from our analysis. This included (i) *Campephilus* woodpeckers that only perform two beats; (ii) some *Picumnus* piculets, which intersperse truncated drumrolls with variable pauses in a single drum display (thus exhibiting irregular acceleration); (iii) and the *Sphyrapicus* sapsuckers, which similarly have irregular acceleration patterns. In some species, both males and females are known to drum; however, the sex-specificity of drumming is unknown for most woodpecker species, and for species in which females do drum there is no known differences in female and male drum performance [18]. Nevertheless, to avoid introducing needless noise to the dataset, we dropped recordings of female individuals from the analysis (*n* = 26, or 3.7% of eligible recordings) [7].

Because drums are acoustically atonal, all measures of drum variation are temporal in nature. We therefore assessed two simple metrics of the drum signal: length (the total number of separate beats in a drum) and speed (the number of beats produced per second). To analyse drums, we measured spectrograms in Adobe Audition CC. Drum length was measured visually, by counting the number of beats in the drum. Because speed characterizes the average rate of drumbeat production over time, we then measured drum duration in seconds by highlighting the time elapsed between the first and last beat of each drum and dividing this by the number of beats. To ensure measurement accuracy, we had a second observer collect the same measurements on a subset of recordings from our database (*n* = 25). When comparing measurements taken by the two observers, we found that both drum length and drum speed were highly repeatable between individuals. The CV_speed_ was 0.9%, whereas the CV_length_ was 0.37%.

All recordings used in this study were gathered from publicly accessible audio archives (Xeno-Canto (http://xeno-canto.org) and the Macaulay Library of Natural Sounds (Cornell University Lab of Ornithology)). We only measured recordings that (i) contained drums from positively identified species, (ii) were of high enough quality that individual beats could be clearly separated both visually and aurally (i.e. successive beats were visible on the spectrogram with blank space between them), and (iii) represented a unique individual, defined by considering any two recordings made at the same time and place to be of the same animal. To further filter our downloaded recordings for consistency, we removed species for which there were fewer than three drums available (*n* = 6). After all these considerations, we collected data from 697 recordings from 164 species, representing an average of 4.7 ± 2.6 (mean ± 1*σ*) individuals per species that each produced an average of 3.4 ± 1.8 drums. From this collection, we also had to remove another 42 species from all analyses, because they were not included in our literature-derived phylogeny due to a lack of sequencing data [22].

### (b) Phylogenetic approach

Broadly, we used phylogenetic comparative methods [31] to investigate how constraint and selection for signal exaggeration interact on a macroevolutionary scale. Specific software and statistical models used follow in each of their respective sections. To ensure our hypothesis testing was not confounded by shared evolutionary history between species, all analyses were phylogenetically controlled based on a maximum clade credibility supermatrix tree, time-calibrated to fossil and biogeographic data, which we derived from Dufort *et al.* [22]. We did not modify the tree structure outside of dropping tips for which we lacked data in the R package phytools [32]. To reconstruct evolutionary gains and losses of drums and drum-like signals (figure 1), we ran a Markov *k*-state one-parameter maximum-likelihood ancestral state reconstruction in phytools, assuming that transitions between each drumming character state (true drum, alternate drum-like signal and no drumming) are equally likely. This was both the simplest and best-fitting model of discrete drum evolution when compared with three alternatives (electronic supplementary material, table S1).

### (c) Constraint and variation

To assess whether morphology constrains drum length and speed across species, we used gathered morphological data from the literature (see electronic supplementary material, References), augmented with specimens from the National Museum of Natural History (USNM, electronic supplementary material, table S1) in Washington, DC. From these specimens, we took three standardized measurements: wing chord (distance from the wrist to the tip of the longest primary feather on an unflattened wing), tail length (length of the longest rectrix) and tarsus length (length of the tarsometatarsal bone). Wing chord and tail length were both measured with a standard wing rule, whereas tarsus length was measured with analogue calipers. These basic measurements are highly standardized and can be compared between records of live birds and museum specimens, which allows us to combine literature-derived measurements with our own. Nonetheless, we also re-measured a subset of species (*n* = 13) for which we gathered measurements from the literature to ensure that our independent specimen measurements were repeatable, and the two groups were indistinguishable for wing chord (*t* = 0.05, *p* = 0.963), tail length (*t* = 0.91, *p* = 0.374) and tarsus length (*t* = 0.86, *p* = 0.401). In addition to wing, tail and tarsus length, we also gathered individual body mass records to use in our analysis, incorporating both the existing literature, as well as USNM specimens for which mass at collection was recorded.

Because our statistical models rely on using a single independent variable, we ran a phylogenetic principal component analysis (pPCA) to reduce morphological data into a single index of size. We took this approach because little is known about woodpecker allometry and PCA allows for convenient computation of a single variable that represents variation among multiple traits. Using pPCA allowed us to control for non-independence due to relatedness [33]. We did this pPCA in phytools [32], using a *λ* model of continuous character evolution [34]. All variation in our four body size variables was explained by four pPCs with pPC1 accounting for 81.4% of the total variation (electronic supplementary material, table S2), so we adopted pPC1 as our index of body size.

To evaluate how PC1 predicts signal exaggeration, we ran quantile regression using the quantreg package in R [35]. Quantile regression tests for a predictive relationship between variables at any specified point in the response variable's (speed or length) distribution (or quantile, *τ*). We use this approach instead of ordinary least-squares (OLS) regression, because constraints on complex animal displays typically appear as a triangular distribution instead of a one-to-one trade-off [36,37]. Because we were interested in the existence of an upper boundary to the distribution between body size and drum speed, we primarily examined models at *τ* = 0.9 and *τ* = 0.8 (i.e. the 90th and 80th percentiles of drum speed and length, respectively), which serves as an unbiased test for the existence of limiting factors [36,37]. However, we ran an entire series of models across the whole distribution to ensure our results were robust to quantile selection (see the electronic supplementary material, appendix A). Because the ‘quantreg’ package cannot internally control for phylogeny, we adopted methodology used previously [38] to run a second set of models that did account for relatedness among species. To do this, we first calculated phylogenetic independent contrasts (PIC) [31] between body size and drum speed or length in the R package ‘caper’, and verified contrast standardization using the package's built-in diagnostic tools [31,39]. PICs are transformed values that account for relatedness among species, and thus introduce a conservative measure of phylogenetic control to the analysis. They can then be supplied to a statistical model and tested to infer how the original traits evolved, as long as the model is forced through the OLS origin [38,40]. Because we ran multiple models, we controlled the false discovery rate on all *p*-values [41].

Given that constraint should limit standing variation in a phenotype, we also compared coefficients of variation (CV = *σ*/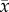) in drum length and speed across species on the within-individual and between-individual scales. In other words, each species has two CV values for each variable (four values total). We restricted this analysis to species that had at least three drums each from three individuals, a sample that encompassed an average of 15.9 ± 1.3 drums from 5.7 ± 0.36 individuals per species. Within-individual CV for a species is the average of each individual's CV across different drums, while between-individual CV (i.e. the species-wide CV) is calculated using the grand mean and standard deviation. To compare variation in drum speed and length across, the woodpecker phylogeny [22], we used a phylogenetic paired *t*-test in phytools [32], because the two samples are dependent and the test controls for relatedness between species. Note that despite its name, the phylogenetic *t*-test is a non-parametric analysis that does not assume normality [42].

### (d) Sexual selection

To test how signal design reflects differences in sexual selection across species, we used SSD as a proxy measure [29,30,43]. SSD is a common index of sexual selection (typically by male–male competition) in many animals, and SSD in birds reflects both direct (i.e. through contests and combat) and indirect (i.e. through territoriality, limited access to mates, extra-pair copulations, etc.) competition among males for mating opportunities [29,30]. We used body mass as our sex-specific size index, which is a strong indicator of overall body size on a broad taxonomic level. After Lisvelend *et al*. [43], we computed SSD as the per cent size difference between male individuals and the population average. As described above, we sourced mass data from the existing literature of either live or recently collected (but not preserved) specimens identified to the species and sex level (see electronic supplementary material, References). Only species with sex-specific mass data for multiple individuals (i.e. *n* > 2 for each sex) were included in the analysis (*n* = 83), where we calculated SSD as the difference between male and female mass divided by the species average mass (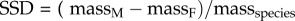). In this way, an SSD equal to 0 indicates a species where males and females are identical in body mass, while species with more positive SSD scores have larger males than females and species with more negative scores have larger females than males.

To test how SSD predicts drum length and speed, we ran phylogenetic generalized least-squares (PGLS) analyses in which we also included body size as a random factor to control for multicollinearity. This is due to the potential for overall size to influence not only signal design, but also SSD itself [30]. We controlled for relatedness between species by using the maximum-likelihood estimation of Pagel's [34] coefficient of relatedness *λ*, which models trait evolution under modified Brownian motion (BM), where 0 ≤ *λ* ≤ 1 and *λ* = 1 reflects full BM. As such, a lower *λ* estimate reflects a trait that evolves more independently of phylogenetic relatedness alone. Again, we report *p*-values that have been corrected for multiple comparisons [41].

## 3. Results

We measured drums and obtained morphological data for 122 species in our phylogeny [22], which exhibit a wide range of values for both drum speed and length (figure 1). Drums or drum-like signals are used by nearly all species in the family, with few transitions among signal states supported by our Mk1 ancestral state reconstruction (electronic supplementary material, table S3).

**Figure 2.**
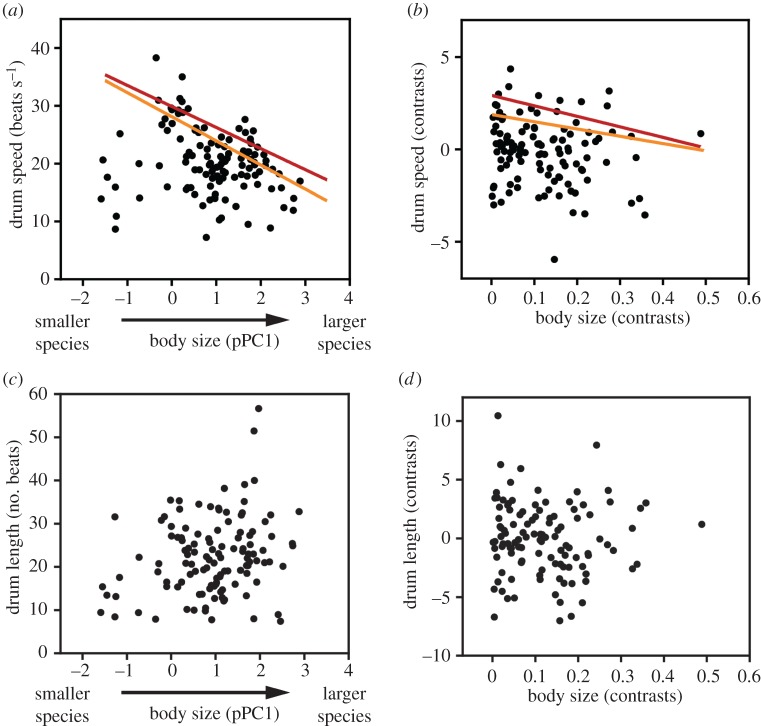
The relationship between body size and drum speed, derived from both raw values (*a*) and phylogenetic independent contrasts (PIC; *b*). Highlighted portions represent the 90th (red; *τ* = 0.9) and 80th (orange; *τ* = 0.8) speed quantiles, at which there is a statistically significant (*p* < 0.01 after correction for multiple testing) trade-off between size and speed. The quantile regression lines appear in solid red and orange for their respective quantiles of *τ* = 0.9 and *τ* = 0.8. Below are body size and drum length from both raw data (*c*) and contrasts (*d*).

**Figure 3.**
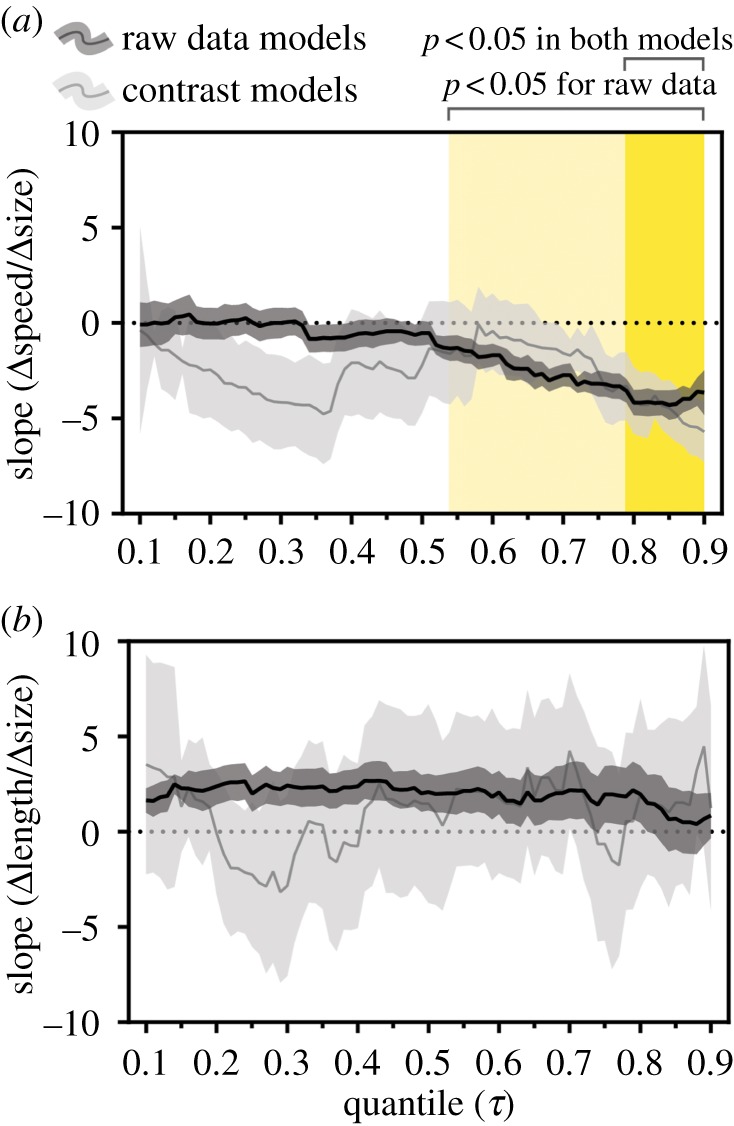
Multimodel results for quantile regressions run on both raw data (black line; ±s.e. in dark grey) and phylogenetic independent contrasts (grey line; ±s.e. in light grey) on the relationship between body size and drum speed (*a*) or drum length (*b*). Each point along a line represents a single model's slope estimate at a different quantile *τ* of the response variable (speed or length). The regions highlighted in yellow indicate significantly non-zero slope estimates (*p* < 0.05) after correction for multiple testing.

We first aimed to test whether drum speed and length are differentially constrained by morphology. Indeed, the relationship between body size (pPC1) and drum speed formed a triangular distribution (figure 2*a*). More importantly, body size negatively predicted drum speed at the 90th (*t* = 3.59, *p* = 0.002) and 80th (*t* = 1.61, *p* = 0.042) speed quantiles. This was also true for the 90th (*t* = 3.14, *p* = 0.002) and 80th (*t* = 6.33, *p* < 0.0001) regression quantiles of PIC data (figure 2*b*). Across the entire speed distribution, our models consistently provided negative best-fit slope values, although these were only significantly non-zero down to the 79th quantile for PIC data (figure 3*a*).

Meanwhile, we found no predictive relationship between body size and drum length on either the raw species values (figure 2*c*) or PIC data (figure 2*d*). This held true across the entire drum length distribution, where no single model had a significantly non-zero slope after controlling for multiple testing (figure 3*b*), where no single model had a significantly non-zero slope after controlling for multiple testing. Combined with the trade-off between size and speed at the uppermost speed quantiles, these results support the idea that speed and length are differentially constrained signals in the complex drum display.

**Figure 4.**
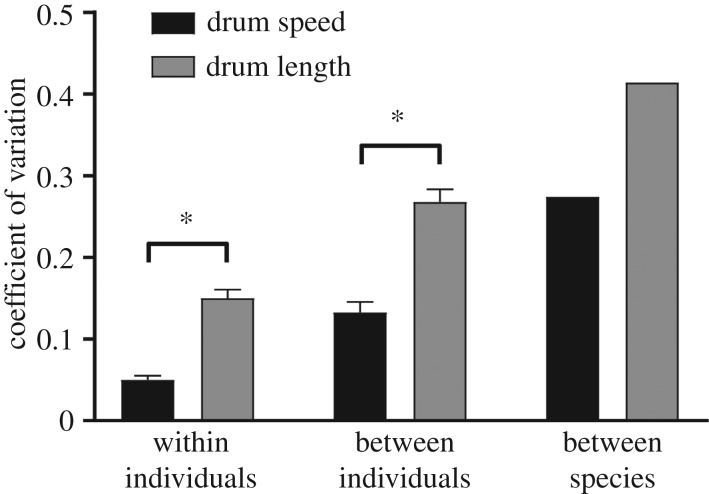
Variation (CV = σ/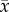) in drum signal structure on multiple scales. Within-individual variation corresponds with speed and length variation within all of a single individual's recorded drums, averaged across species. Between-individual CV characterizes variation within an entire species. Finally, between-species CV is the single value for the entire dataset, describing differences across the woodpecker radiation. Brackets with asterisks (*) mark statistically significant differences between speed and length variation on within-individual (*p* < 0.0001) and between-individual (*p* = 0.0097) scales via phylogenetic paired *t*-tests.

To determine whether differences in constraint are also connected with standing variation, we compared the coefficient of variation (CV) between drum speed and length on multiple scales (figure 4). We found that size-constrained drum speed is less variable than length on both within-individual (*t* = 5.321, *λ* = 0.264, *p* < 0.0001) and between-individual scales (*t* = 2.652, *λ* = 0.627, *p* = 0.0097).

**Figure 5.**
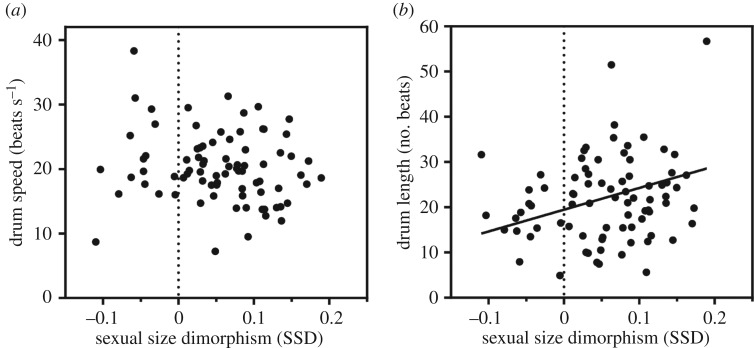
SSD as it predicts species averages for drum speed (*a*) and length (*b*). The vertical dotted line indicates SSD = 0, where males and females are identical in body mass; species with negative SSD have females larger than males and positive SSD indicates males larger than females. The solid line represents a statistically significant relationship between SSD and drum length (*λ* = 0.875, *p* = 0.003).

Finally, we assessed how sexual selection operates within the phenotypic space constrained by body size. We therefore tested how SSD predicts species variation in drum length and speed. We found that species variation in SSD did not predict differences in drum speed (figure 5*a*; *F*_2,77_ = 1.719, *λ* = 0.822, *p* = 0.975). We did, however, uncover a significant positive relationship between SSD and drum length (figure 5*b*; *F*_2,77_ = 5.966, *λ* = 0.875, *p* = 0.0031). Thus, species in which males are larger than females tend to produce longer drums, compared with species where females are the same size or larger than males. To ensure this effect was not confounded by body size, we verified that it had neither a significant effect on speed (*F*_2,77_ = 0.63, *p* = 0.428) nor an interaction with size (*F*_2,77_ < 0.0001, *p* = 0.996).

## 4. Discussion

Here, we demonstrate how constraint and sexual selection differentially influence multiple signalling traits in woodpecker drums, which are complex physical displays. As such, we find that species with the fastest drums undergo a robust trade-off between drum speed and body size. However, this relationship does not exist for drum length. At the same time, our data show that SSD (a proxy for the intensity of sexual selection in birds [29,30]) is positively correlated with drum length, but not drum speed. These effects are also reflected in suppressed standing variation in speed, but not length. Therefore, our findings collectively demonstrate two novel consequences of differential constraint on display evolution: (i) sexual selection preferentially exaggerates a signal that is unconstrained by body size and (ii) constraint is associated with reduced standing variation. Altogether, these findings support a model where displays evolve in a modular fashion, with length and speed changing independently in response to interactive selection regimens [10].

This idea that complex displays are shaped by multiple evolutionary forces was first supported by studies of Trinidadian guppies, where male ornamentation varying in both colour and pattern is shaped not only by sexual selection, but also local predation pressure and visibility [2,4]. Similarly, displays used by túngara frogs (*Engystomops pustulosus*) that function both in male–male competition and mate attraction consist of multiple acoustic elements combined with visual stimuli [13]. One reason such complex displays may have evolved lies in the significance of multiple messages: for each signal incorporated into the display, either new or redundant information can be encoded to mediate social interactions [44]. The different components of this multifaceted display are each subject to distinct selection pressures, in which sexual selection operates around constraints imposed by morphology, predation and the signalling environment [6,13]. When the components of these complex displays arise independently, the result is a modular display in which individual modifications to signal parts result in functional shifts of the display as a whole [10]. Our current work therefore suggests that the consequences of modular display evolution can be borne out on a macroevolutionary scale, whereby species-level display divergence emerges from complex intersections between morphology and potent sexual selection.

Why is drum speed constrained by body size, while drum length is not? The nature of selective constraint is likely anchored in the biomechanical mechanisms of the signal's production. A drumming woodpecker is typically modelled as a modified angular rod [24,25], wherein proportional increases in body size increase the linear distance that an individual must traverse to strike the drumming substrate (electronic supplementary material, figure S1). It therefore becomes more difficult for larger species to drum as fast as smaller species. Interestingly, this framework is similar to vocal constraint models of birdsong, in which vocal tract morphology can influence song pace characteristics on both proximate and evolutionary timescales [7–9]. In reality, however, an individual woodpecker is more than a simple rod [25], and species variation in body size is not always a matter of proportional scaling. Following this logic, it is tempting to imagine that some species might circumnavigate the speed-size trade-off if they undergo proportional modifications to certain body regions that would make it easier to produce high-speed drums. For example, an increase in bill length may facilitate high-speed drumming by decreasing the distance an individual has to travel to produce every beat in a drum. However, such evolutionary ‘solutions’ to the issue of fast drumming must be considered cautiously; for instance, this putative method of overcoming size constraints by evolving a longer bill is not likely the case, as most of the species that produce fast drums for their body size are in fact short-billed species from the genera *Picoides*, *Veniliornis* and *Celeus*. Additionally, morphology is under strong ecological selection for foraging efficiency in both woodpeckers and other bird species [45,46], which likely takes precedence over sexual selection for display elaboration [8,9,46]. Regardless of which biomechanical mechanism is responsible, our results clearly demonstrate its phenotypical consequences on a macroevolutionary scale: there is a limit to how fast a given species can drum, and standing variation in drum speed is reduced relative to drum length.

Even though we find that size does not constrain drum length, this does not necessarily mean the signal is phenotypically limitless. Instead, we suspect that drum length is constrained by factors other than body size that may influence signal function. Of the many factors that may constrain display length, likely candidates include muscle performance and/or fatigue resistance [47]. A growing body of work suggests that muscle function is a critical determinant of display performance linked to elaborate body and limb movements, which can be reflected in morphological differences between species [48]. Indeed, most woodpeckers maintain hypertrophied *longus colli* neck muscles, which are thought to be the major actuators of drum behaviour [49]. Although large muscles may aid other behaviours as well as drumming, this local hypertrophy should support the robust head and neck movements necessary to produce an effective drum. If these physiological modifications do indeed underlie how long an individual can continue drumming, then drum length may in turn function as an index of individual vigour, or the inherent ability to repeatedly perform challenging and costly manoeuvres [50].

Because we find that SSD positively predicts species variation in drum length, our data suggest that sexual selection shapes woodpecker drums by preferentially exaggerating this component unconstrained by body size. In some ways, these data challenge the long-held notion that displays must be unilaterally honest indicators of individual quality, as constraints are thought to enforce signal honesty [21]. However, sexual selection for display behaviour is not always so utilitarian—some displays evolve via non-adaptive female choice, where mate choice favours display traits that convey no honest signalling advantage [15,28]. In fact, variation itself is important in the displays of many species, as it provides the basis for signal modulation across different social contexts [16,19]. This is even true for woodpeckers; for example, wild downy woodpeckers (*Dryobates pubescens*) respond more aggressively to longer drums [18], and will increase drum speed when confronted with a fast-drumming intruder [19]. Our results suggest that the same phenomenon plays out at a macroevolutionary scale with respect to length, but not speed. This might be because the downy woodpecker's drum speed (16 beats s^−1^) is relatively slow, falling below the median speed (50th quantile) of 19.7 beats s^−1^ among all woodpeckers. This means that drum speed can still be modulated despite the difference in variation between length and speed, at least for species occupying lower portions of the distribution. It also serves as a reminder that the trade-off between size and speed only appears to influence macroevolutionary variation among the fastest-drumming species. Thus, although the overall phenotypic space for potential elaboration is restricted, there are numerous other factors that influence speed exaggeration [37]. For instance, selection for species recognition may influence drum speed, as most regions support multiple woodpecker species that use drum speed to distinguish between different species [19,26]. Models of arbitrary mate choice or competitive effectiveness may also be among the numerous alternate explanations [27,28].

In conclusion, our results illustrate a trajectory for sexual selection to exaggerate complex displays under constraint: by selectively acting on the unconstrained components of a signal that are otherwise best suited to accomplish its adaptive purpose. The result is a modular display, which is generated when sexual selection sequentially elaborates multiple signal components that are each subject to a unique evolutionary trajectory. In this case, sexual selection preferentially exaggerates a labile element for territorial defence, while another different element remains relatively rigid. Thus, by testing hypotheses at the intersection of constraint and elaboration, we can begin to elucidate the mechanisms by which dynamic selection regimens yield remarkable phenotypical diversity across an entire animal family.

## Supplementary Material

Appendix A

## Supplementary Material

Supplementary Tables & Figures

## Supplementary Material

Supplementary Data File

## Supplementary Material

Supplementary Recording List

## Data accessibility

Morphological data and recording accession numbers are accessible via the electronic supplementary material, and additional drum data are available upon request from the corresponding author.
