# Supplementary material for: Macroevolutionary patterning of woodpecker drums reveals how sexual selection elaborates signals under constraint
Source: Proc Biol Sci. 2018 Feb 21;285(1873):20172628. doi: 10.1098/rspb.2017.2628 (PMC5832706; doi:10.1098/rspb.2017.2628)
Supplement: Appendix A [file rspb20172628supp1.pdf]

Appendix A. Quantile Regression Supplement

Quantile regression vs. ordinary least squares regression

Quantile regression is similar to an ordinary least squares (OLS) regression in that it fundamentally tests for a predictive association between two continuous variables. However, OLS regression is a parametric model that only evaluates relationships based on the response's predicted mean. This is often impractical for complex behavioral and ecological datasets, because relationships between variables can change across the distribution of those variables. For example, in many birds that perform trilled vocalizations, there exists a performance trade-off between each note's frequency bandwidth and the rate at which notes are produced (trill rate)—but this trade-off is only apparent for individuals that produce songs at the upper portion of the bandwidth or trill rate distribution, and is thus not detected by OLS regression (Podos 1997, 2001; Wilson *et al.* 2014). By contrast, quantile regression assesses predictive relationships at any quantile (*i.e.* percentile) of the response variable, and is also not reliant on parametric assumptions (Koenker & Machado 1999; Cade, Brian S.; Noon 2003). This makes quantile regression a powerful and flexible tool for exploring complex relationships between variables that would not otherwise be captured by OLS regression. Indeed, this model has become a mainstay in ecological studies (reviewed in Cade, Brian S.; Noon 2003). (reviewed in Cade, Brian S.; Noon 2003; Wilson *et al.* 2014)

Quantile regression in animal behavior

Much work attempting to understand the evolution of complex animal displays concerns the existence of signaling trade-offs and constraints on display production. Historically, testing for these trade-offs relied on generating a plot of two continuous variables (if applicable, the

predictor is the hypothesized constraint and the response is the biologically relevant signaling trait) and testing for the existence of a triangular distribution (Fig. A1). As the name implies, triangular distributions look like a right triangle, where the lowest X values span a wide range of Y values, and vice versa. However, at the upper limits of the distribution, there is a hard diagonal boundary above which values do not appear (see Podos 1997 for examples). The existence of this boundary suggests a biological reality in which the predictor variable restricts the response variable, but only at its highest values (*i.e.* in the upper quantiles).

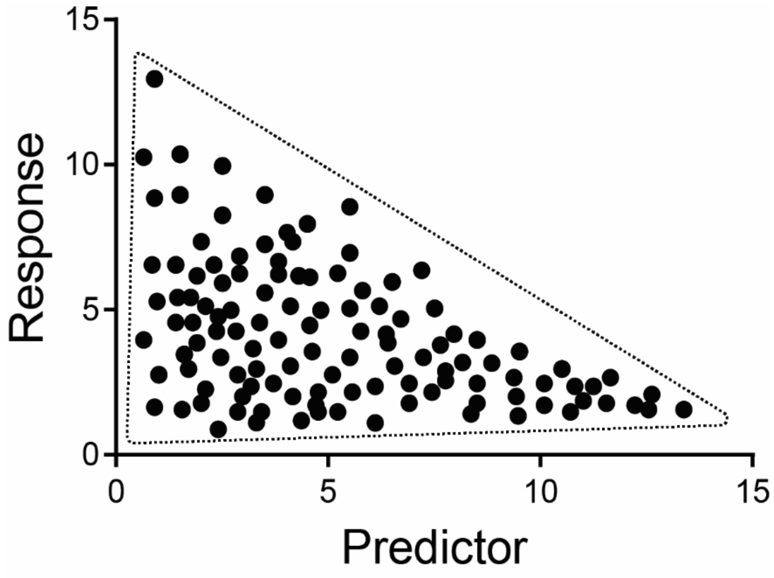

Figure A1. A typical triangular distribution, derived from simulated data. The lower quantiles of the predictor variable have a wider range of response values, and vice versa, and there is a firm upper limit to response values given the predictor. The “hypotenuse” of this right triangle distribution forms the trait boundary that most researchers studying animal phenotypes are interested in testing, but would not be detected by OLS regression.

Early attempts to test for the existence of this boundary relied on a modified OLS regression approach called upper-bound regression, in which researchers binned the predictor variable with various approaches. From each bin, only the point with the highest response value would be supplied to the OLS model, effectively generating a linear regression for the upper quantile of the distribution. However, upper bound regression is a somewhat biased approach

because it relies on the observer to choose how to bin the predictor variable, which in turn influences the model's results (Wilson *et al.* 2014). The use of quantile regression as an alternative has since gained traction for animal behaviorists, where testing for relationships at the 90th quantile (τ=0.9) can detect upper bounds in an unbiased fashion (e.g. Olsen *et al.* 2013; Wilson *et al.* 2014; Davidson *et al.* 2017). However, other studies of animal behavior highlight the importance of investigating multiple quantiles to uncover hidden trends in the data (Busch & Mehner 2012; Chamaillé-Jammes & Blumstein 2012; Jovani *et al.* 2016). This can be done easily in R using the 'quantreg' package (Koenker 2013), where the user can specify the range of τ values. Moreover, quantile regression becomes more sensitive to outliers when testing for relationships at extremely high or low portions of the distribution, so a single test at τ=0.9 may potentially be spurious.

Quantile regression on body size and drum characteristics

Although our main focus was establishing whether or not a statistically significant upper boundary (at τ=0.9) existed between our phylogenetic principal component score of body size (pPC1) and drum speed, we also ran an iterative series of quantile regressions for $0.1 \leq \tau \leq 0.9$, where we adjusted τ at increments of 0.01. Slope estimates of the relationship between pPC1 and drum speed or length indeed supported a model where only species that drum in the upper speed quantiles ($\tau \geq 0.8$) exhibit a significant negative relationship between size and drum elaboration (Fig. A2). This suggests that drum speed is not only constrained by body size for the fastest-drumming species, but instead that morphological constraints on drum speed extend deeper into the distribution.

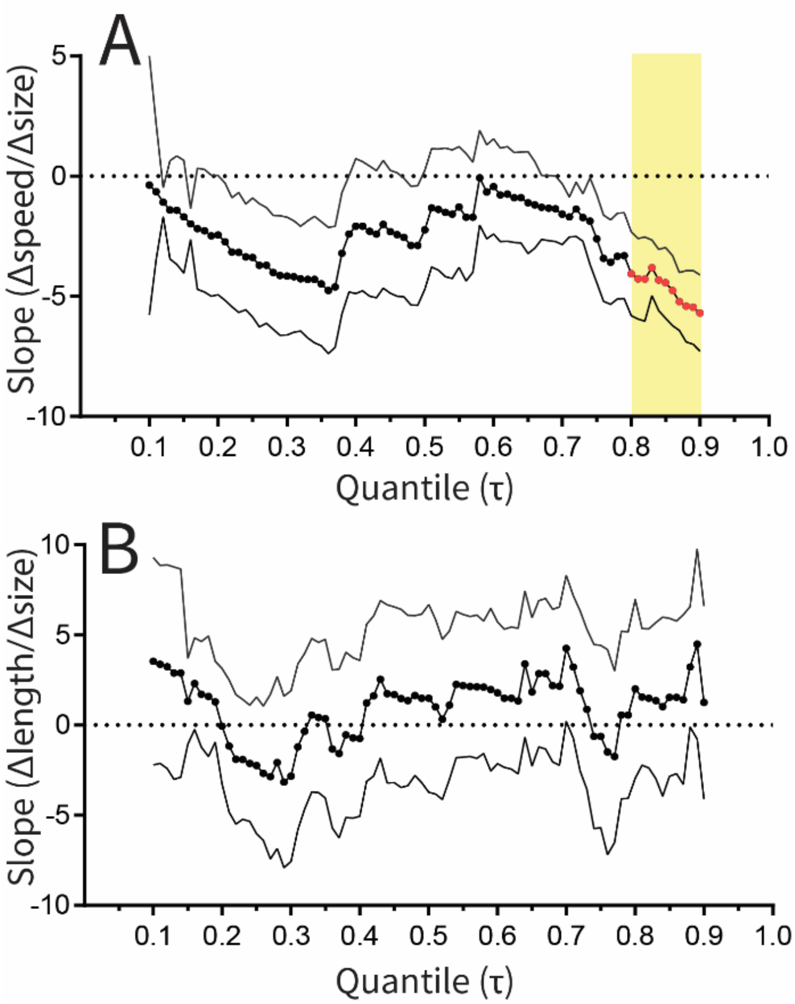

Figure A2. Quantile regression model estimates (± standard error) of the linear relationship (slope) between phylogenetic independent contrasts of body size (pPC1) and drum speed (A) or length (B) across different quantiles 0.1≤τ≤0.9. Models were run iteratively, increasing τ by increments of 0.01. Red points in the yellow highlighted region designate models in which the slope was statistically significant ($p>0.05$ after correction for multiple testing).

Meanwhile, there was no point in the drum length distribution where body size significantly predicted length elaboration. All τ values between 0.8 and 0.9 maintained a statistically significant relationship between size and speed after correction for multiple testing (Table A1), which ensures that our decision to test and report the trade-off at τ=0.9 was robust to quantile selection.

Table A1. Quantile regression slope estimates for the relationship between phylogenetic independent contrasts of species body size and drum speed at quantiles 0.8≤τ0.9. Statistical inferences are derived from comparing the 95% confidence intervals of slope estimates to the t-distribution to test H_0: slope = 0. All p-values reported have been adjusted to account for multiple testing (Holm 1988). * p<0.05, ** p<0.01, *** p<0.001

**Quantile (τ)**	**Slope estimate**	**t-value**	**p-value**
0.8*	-4.06	-1.61	0.0422
0.81*	-4.26	-1.71	0.0248
0.82*	-4.27	-1.75	0.0294
0.83**	-3.81	-1.54	0.0036
0.84**	-4.31	-1.77	0.0031
0.85**	-4.43	-1.87	0.0069
0.86**	-4.76	-2.04	0.0036
0.87***	-5.21	-2.29	0.0006
0.88**	-5.41	-2.27	0.0021
0.89**	-5.46	-2.37	0.0021
0.9**	-5.69	-2.55	0.0021

Busch, S. & Mehner, T. (2012) Size-dependent patterns of diel vertical migration: Smaller fish may benefit from faster ascent. *Behavioral Ecology*, **23**, 210–217.

Cade, Brian S.; Noon, B. (2003) A gentle introduction to quantile regression for ecologists. *Frontiers in Ecology and the Environment*, **1**, 412–420.

Chamaillé-Jammes, S. & Blumstein, D.T. (2012) A case for quantile regression in behavioral ecology: getting more out of flight initiation distance data. *Behavioral Ecology and Sociobiology*, **66**, 985–992.

Davidson, B.M., Antonova, G., Dlott, H., Barber, J.R. & Francis, C.D. (2017) Natural and anthropogenic sounds reduce song performance: insights from two emberizid species. *Behavioral Ecology*, **84**, E1–E9.

Holm, S. (1988) A simple sequentially rejective multiple test procedure. *Scandinavian Journal of Statistics*, **6**, 65–70.

Jovani, R., Lascelles, B., Garamszegi, L.Z., Mavor, R., Thaxter, C.B. & Oro, D. (2016) Colony size and foraging range in seabirds. *Oikos*, **125**, 968–974.

Koenker, R. (2013) quantreg: Quantile Regression. R package version 5.05. *R Foundation for Statistical Computing: Vienna) Available at: http://CRAN. R-project. org/package= quantreg*.

Koenker, R. & Machado, J.A.F. (1999) Goodness of fit and related inference processes for quantile regression. *Journal of the American Statistical Association*, **94**, 1296–1310.

Liu, Y., Qiu, X., Yu, T., Tao, J. & Cheng, Z. (2015) How does a woodpecker work? An impact dynamics approach. *Acta Mechanica Sinica*, **31**, 181–190.

Olsen, B.J., Greenberg, R., Walters, J.R. & Fleischer, R.C. (2013) Sexual dimorphism in a feeding apparatus is driven by mate choice and not niche partitioning. *Behavioral Ecology*, **24**, 1327–1338.

Podos, J. (1997) A performance constraint on the evolution of trilled vocalizations in a songbird

family (Passeriformed: Emberizidae). *Evolution*, **51**, 537–551.
Podos, J. (2001) Correlated evolution of morphology and vocal signal structure in Darwin's finches. *Nature*, **409**, 185–188.
Wilson, D.R., Bitton, P.-P., Podos, J. & Mennill, D.J. (2014) Uneven sampling and the analysis of vocal performance constraints. *The American Naturalist*, **183**, 214–228.
