# Supplementary material for: Macroevolutionary patterning of woodpecker drums reveals how sexual selection elaborates signals under constraint
Source: Proc Biol Sci. 2018 Feb 21;285(1873):20172628. doi: 10.1098/rspb.2017.2628 (PMC5832706; doi:10.1098/rspb.2017.2628)
Supplement: Supplementary Tables & Figures [file rspb20172628supp2.pdf]

Supplementary Figures

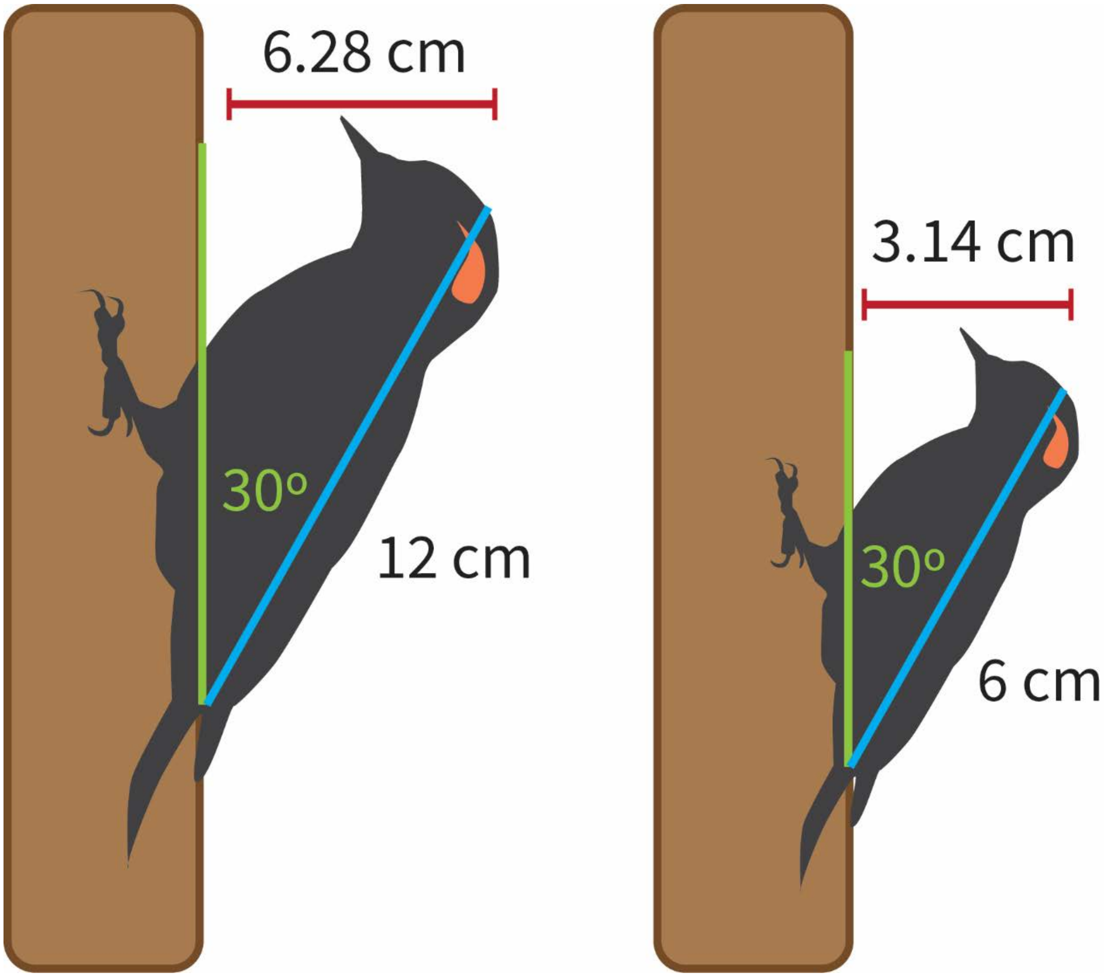

Figure 1S. Schematic of the hinged woodpecker drumming model (simplified from (Liu *et al.* 2015), demonstrating how increasing body size may theoretically affect drum speed performance. Because a woodpecker drums by rotating the body, its movement can be modeled using the angular velocity equation $d=r\theta$, (d = linear distance to travel, r = body length, and θ = angular distance, in radians). Given the same body position angle, which determines angular distance, a larger species has to traverse a greater distance to strike the resonate surface than a smaller species does.

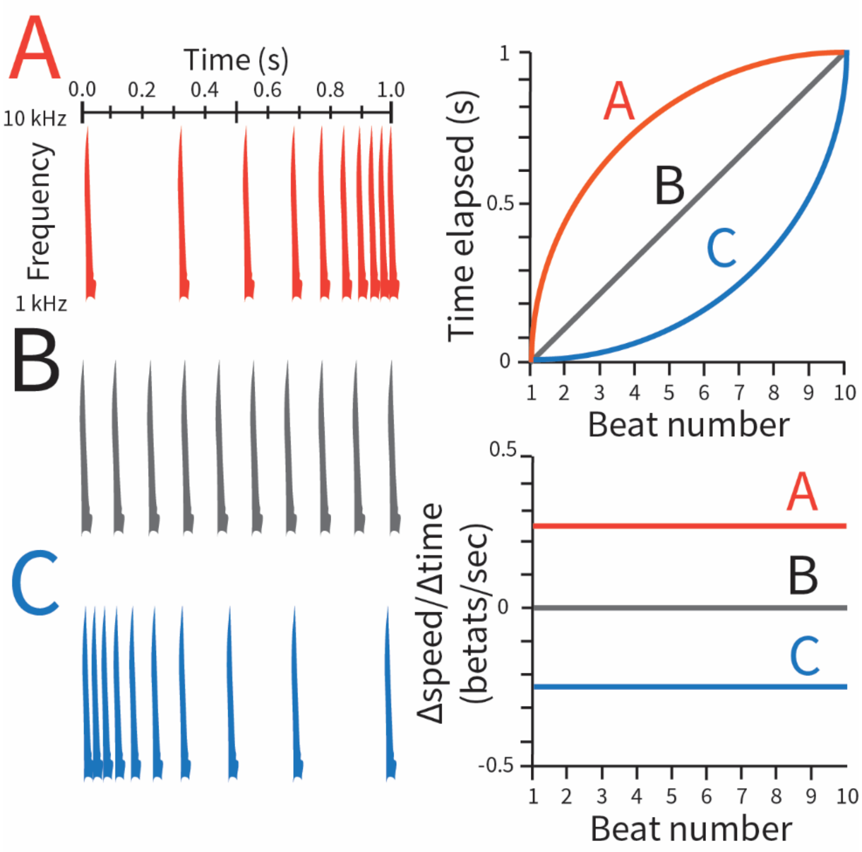

Figure 2S. Spectrograms of theoretical (but representative) woodpecker drums are drawn, depicting the three types of drums considered to be "typical" for the purposes of this study: accelerating (A), constant (B), and decelerating (C). Although each of these drum types has a different acceleration value (*i.e* instantaneous drum speed changes differently over time for each), acceleration does not change over time. Therefore, despite differences in speed changes across these drums, each of them is characterized as having 10 beats per second. Although the majority of woodpecker species produce drums that conform to one of the three patterns above, some species drum with shifting acceleration rates, where longer pauses are interspersed with a short series of fast beats. Because such erratic drums consist of a combination of fast and slow elements, average speed metrics are ineffective as a biologically relevant descriptor of cadence.

Supplementary Tables

Table 1S. Log marginal likelihood scores for discrete models of three drumming character states (0=no drum, 1=typical drum, 2=irregular "drum"). Transitions were generally uncommon (see Fig. 1), so introducing additional parameters to the model did not improve its fit over the simplest equal rates (ER) model.

model type		log marginal lik
Complete ER	all (aka null)	-60.13634
All variable	none	-66.149
Gains=Loss	01=10, 02=20, 12=21	-63.992
Gains=Loss, 12 varies	01=10, 02=20	-62.634

Table 1S. Accession numbers for all museum specimens measured as part of this study. Specimens were measured on location at the Smithsonian National Museum of Natural History (USNM, Washington, DC). † Denotes species included to validate measurements; specimens with an asterisk (*) had mass recorded at the time of collection.

**Species**	**Female Specimens (USNM#)**	**Male Specimens (USNM#)**
Celeus lugubris	N/A	609513*, 16399, 284581, 584582
Celeus ochraceus	38856, 516031, 120983	37042
Celeus torquatus	622347*	16681, 514785, 625423*
Celeus undatus	589337*, 516272, 51622, 625154*, 627579*	627042*
Celeus elegans†	627578*, 625152*, 637063*, 65153*	651994*
Colaptes punctigula	373130, 401861, 392497, 411106, 368963	401855, 401864, 401859, 41107, 425983
Colaptes atricollis	159794, 95859	N/A
Colaptes melanochloros†	516834, 516848, 516846, 516831, 516829, 516828, 516842, 516863, 516860, 516830	16715, 18280, 13782, 18977, 516855, 21270, 16717, 516844, 516845, 516838
Colaptes (auratus) cafer	269441, 590038, 270792, 270791, 363620, 157617, 201488, 157606, 115755	586220, 59004, 158064, 142320, 132787, 140490, 140491, 140488, 140492, 113655
Colaptes rivolii†	41851, 411095, 373089, 401852, 392563, 392559, 373091, 373059	373090, 373093, 411093, 392556, 392558, 411094, 392562
Chrysocolaptes xanthocephalus	192593, 315198	192592, 315199, 233500, 315197, 315200
Chrysocolaptes guttacristatus†	332810, 172995, 452104, 473925, 452691, 313280, 278360, 313279, 313282, 360734	306889, 308074, 451681, 308574, 324146, 330308, 278363, 451535, 451534, 332812

Chrysocolaptes validus†	182315, 333721, 160913, 333723, 333722, 483368, 160201	181075, 160191, 160193, 178991, 310882, 178948, 333719, 160192
Chrysophlegma mentale	324215, 169770, 180973, 178943, 179473, 333754, 169769, 160205	333757, 333758, 333756, 333755, 310871, 160202, 306921, 172991
Chrysophlegma flavinucha	408167, 459403, 333760, 336303, 450255	585769, 335554, 391009, 585273, 459404, 452667
Dryocopus fulvus	218410, 249128, 248415, 248419, 248416	249132, 248423, 249655, 248422, 248413
Melanerpes santacruzi	89992, 646700*	361309, 361308, 154063, 154972, 154962, 349555, 646760*
Melanerpes flavifrons†	115181, 516032, 264923	264922, 115181, 177655, 14160, 145605, 109770
Melanerpes cruentatus	325963, 255873, 389296, 326693, 326692, 587550, 326690	586339, 586340, 326691, 326688, 326689, 22846, 326694, 325962
Picumnus rufiventris	609377	533537
Picumnus nebulosus	635844	630511*, 635843*
Piculus flavigula	586335*, 572532, 368214	637002*, 625417*, 610186*, 625151*, 625150*
Dendropicos goertae	462707, 426706, 214137, 214136	552866, 335028, 460400, 214138, 213461
Dendrocopos hyperythrus	519773, 296315, 314078, 408193, 296314, 296312	312038, 336299, 332840, 31479, 336985, 312042
Dendrocopos darjellensis†	408192, 306098, 296301, 408189, 296302	314071, 536087, 408187
Dendrocopos analis	218392, 218393, 218896	218893, 218390, 219322
Dendrocopos macei†	408192, 306098, 296301, 408189, 296302	585276, 390997, 408200, 145574, 408197, 449442, 584468, 585279, 449444, 585169, 585168, 390209, 449443
Dendrocopos syriacus†	37715, 522800*, 536655*,	536656*, 522799*
Dendrocopos major†	639859*, 639731*, 639787*, 639739*, 639765*	640899*, 640332*, 640941*, 640535*, 640898*, 640204*
Dendrocopos noguchii	359399, 385205	385204
Picoides canicapillus	450318, 331895, 336833, 150311, 452687	451928, 311481, 334464, 450306, 450313
Picoides kizuki	633353*, 91426, 91334, 109399, 518433, 125235	633364*, 109398, 91427, 91333, 526515, 526513
Veniliornis lignarius	284964*, 485546, 352639	264926, 48997, 485545, 14353
Veniliornis mixtus	631247*, 49417, 255229, 52982, 630298*, 631317*	631248*, 5585
Veniliornis frontalis	284863*	645289
Veniliornis affinis†	455292	515930, 515929, 368223, 368222, 515928, 455291
Veniliornis callonotus	644057, 526227, 536766, 305153, 305154	527831, 54098

Veniliornis dignus†	426032, 426029, 401854	426026, 426030, 446458, 426027, 426024
Veniliornis nigriceps	446462	325845
Veniliornis spilogaster	635950*, 645387*	516867, 284609, 516892, 516872, 635950, 516874, 645387
Veniliornis sanguineus	625426*, 625427*, 625947*, 626096*	55212, 625424*, 625425*
Veniliornis cassini	625429*, 625155*, 622278	131921, 605764, 621747*, 625428, 637021
Dendropicos namaquus	455179, 46124, 527346*, 527347*	546106, 433166, 527348, 460125, 437270, 546107
Dinopium rafflesii	14137	483369*, 385204, 100237, 180479, 145582

Table 2S. Loadings for four phylogenetic principal components (pPCs) run on correlated measures of species morphology: mass, wing chord, tail length, and tarsus length. We only used pPC1 in subsequent analyses because it accounted for 87.4% of variance in the data (from unidirectional loadings of all variables) while still accounting for the effects of correlated evolution due to shared history between species.

Variable	*pPC1*	*pPC2*	*pPC3*	*pPC4*
Mass	0.811	0.514	-0.212	0.178
Wing chord	0.32	-0.179	0.011	-0.93
Tail length	0.405	-0.835	-0.226	0.298
Tarsus length	0.274	-0.081	0.951	0.121
Variance explained	0.874	0.079	0.029	0.018
Cumulative variance explained	0.874	0.953	0.982	1
Eigenvalue	11.54	1.043	0.3817	0.2325

Supplementary References: Literature-derived Morphological Measurements

Winkler H, Christie DA, Nurney D. 1995 *Woodpeckers: an identification guide to the woodpeckers of the world*. Boston: Houghton Mifflin.

Short LLJ. 1970 Notes on the habits of some Argentine a Peruvian woodpeckers (Aves, Picidae). *Am. Museum Novit.* , 1–36.

Reinert BL, Pinto JC, Bornschein MR, Pichorim M, Marini MÂ. 1996 Body masses and measurements of birds from southern Atlantic Forest, Brazil. *Rev. Bras. Zool.* **13**, 815–820. (doi:10.1590/S0101-81751996000400001)

Dunning Jr JB. 1992 *CRC handbook of avian body masses*. CRC press.

Haverschmidt F. 1948 Bird weights from Surinam. *Wilson Bull.* **60**, 230–239. (doi:10.2307/4157709)

Lislevand T, Figuerola J, Szekely T. 2007 Avian body sizes in relation to fecundity, mating system, display behaviour, and resource sharing. *Ecology* **88**, 1605. (doi:10.1890/11-1341.1)

Haverschmidt F. 1952 More bird weights from Surinam. *Wilson Bull.* **64**, 234–241. (doi:10.1080/00306525.1969.9634329)

del Hoyo J, Elliot A, Sargatal J. 2002 *Handbook of the Birds of the World, Volume 7: Jacamars to Woodpeckers*. Lynx Edicions.

Gorman G 2014 *Woodpeckers of the World*. Firefly Books.
